# Supplementary material for: WEE1 inhibitors synergise with mRNA translation defects via activation of the kinase GCN2
Source: Nat Commun. 2025 Oct 9;16:8983. doi: 10.1038/s41467-025-64050-5 (PMC12511557; doi:10.1038/s41467-025-64050-5)
Supplement: Supplementary file 1 — Supplementary Information [file 41467_2025_64050_MOESM1_ESM.pdf]

Supplementary Information

a

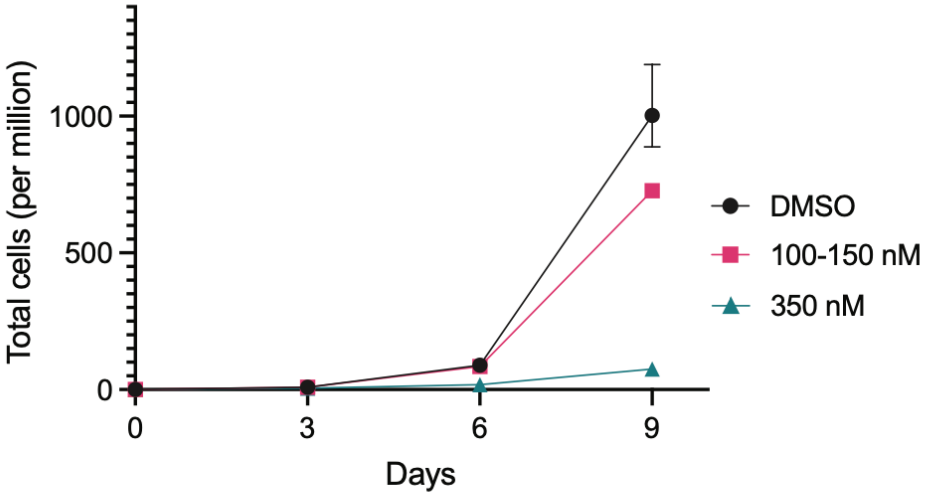

b

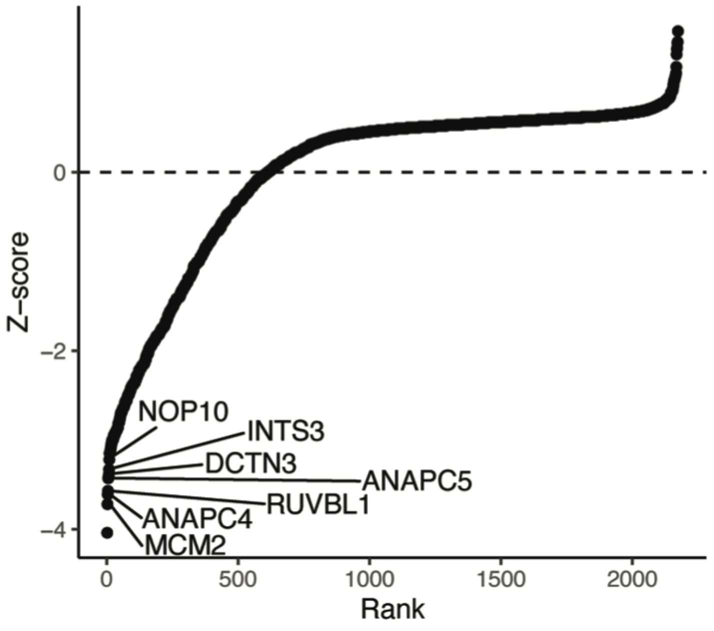

Supplementary Fig.1: CRISPRi screen dosing and essentialome. a Graph showing the growth over time of RPE TP53$^{-/-}$ dCas9-KRAB cells treated with either DMSO or AZD1775. The 100-150 nM AZD1775 arm was treated with 100 nM for days 0-6 and treated with 150 nM on days 6-9 (biological n=2). Graphs are depicted with means ± SD. **b** Graph showing the NormZ score of sgRNAs on day 18 (DMSO treated arm) post-transduction compared to day 3 post-transduction. A handful of essential genes have been labelled to show a decrease of sgRNA representation on day 18 post-transduction. Source data are provided as a Source Data file.

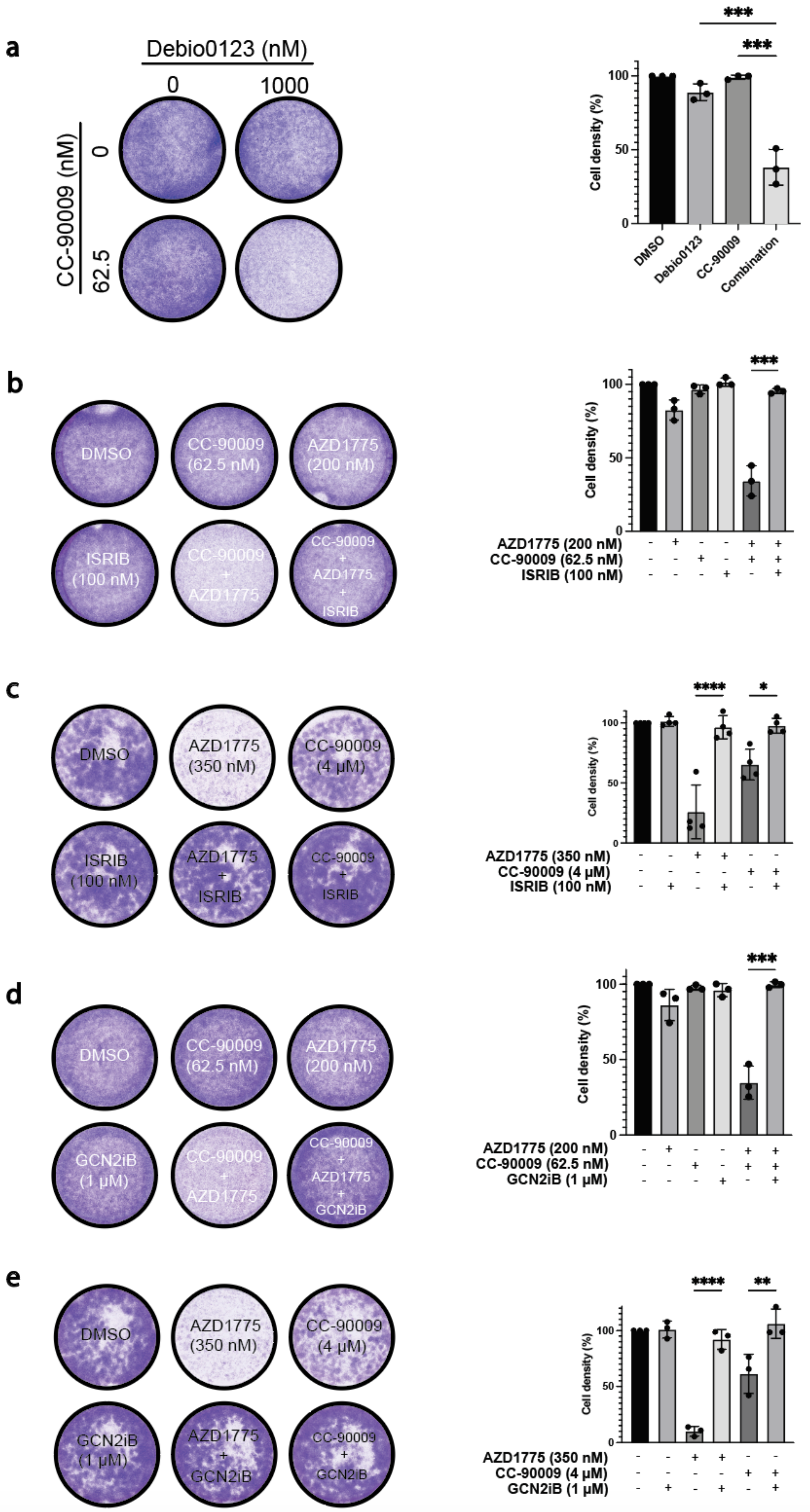
a
Debio0123 (nM)
0
1000
CC-90009 (nM)
0
62.5
***
***
Cell density (%)
100
50
0
DMSO
Debio0123
CC-90009
Combination
b
DMSO
CC-90009 (62.5 nM)
AZD1775 (200 nM)
ISRIB (100 nM)
CC-90009 + AZD1775
CC-90009 + AZD1775 + ISRIB
***
Cell density (%)
AZD1775 (200 nM)
CC-90009 (62.5 nM)
ISRIB (100 nM)
c
DMSO
AZD1775 (350 nM)
CC-90009 (4 µM)
ISRIB (100 nM)
AZD1775 + ISRIB
CC-90009 + ISRIB
****
*
Cell density (%)
AZD1775 (350 nM)
CC-90009 (4 µM)
ISRIB (100 nM)
d
DMSO
CC-90009 (62.5 nM)
AZD1775 (200 nM)
GCN2iB (1 µM)
CC-90009 + AZD1775
CC-90009 + AZD1775 + GCN2iB
***
Cell density (%)
AZD1775 (200 nM)
CC-90009 (62.5 nM)
GCN2iB (1 µM)
e
DMSO
AZD1775 (350 nM)
CC-90009 (4 µM)
GCN2iB (1 µM)
AZD1775 + GCN2iB
CC-90009 + GCN2iB
****
**
Cell density (%)
AZD1775 (350 nM)
CC-90009 (4 µM)
GCN2iB (1 µM)

Supplementary Fig.2: 6 well plate crystal violet assays on the RPE TP53$^{-/-}$ cell line. Representative images and graphs showing the RPE TP53$^{-/-}$ cell line treated with DMSO, WEE1 inhibitors, ISRIB, GCN2iB alone and in combination. Following their respective timepoints, 6 well plates were washed and stained with crystal violet (biological n=3 with exception of c which is n=4). Graphs are depicted with means ± SD, points represent each biological replicate. Statistical analyses were performed using unpaired two-tailed t-test (**b, d)** or one-way ANOVA with multiple comparisons (**a, c, e)** tests, * $p < 0.05$, ** $p < 0.01$, *** $p < 0.001$, **** $p < 0.0001$. **a, b, d** Cells were treated for 72 hours. **c, e** Cells were treated for 6 days. After 72 hours, fresh media and drug was applied. Source data are provided as a Source Data file.

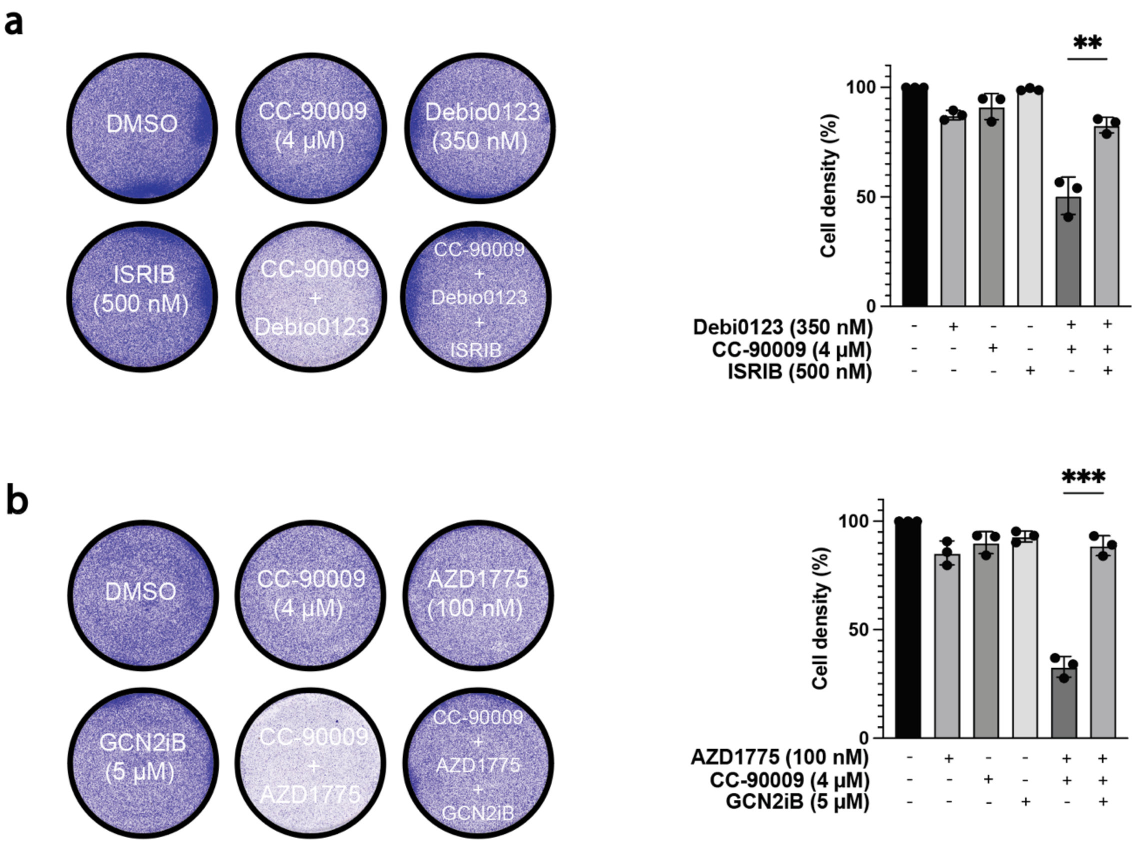

Supplementary Fig.3: 6 well plate crystal violet assays on the HAP1 cell line. a, b Representative images and graphs showing the HAP1 cell line treated with DMSO, WEE1 inhibitors, CC-90009, ISRIB, GCN2iB alone and in combination for 72 hours. Following their respective timepoints, 6 well plates were washed and stained with crystal violet (biological n=3). Graphs are depicted with means ± SD, points represent each biological replicate. Statistical analyses were performed using unpaired two-tailed t-tests, ** $p < 0.01$, *** $p < 0.001$. Source data are provided as a Source Data file.

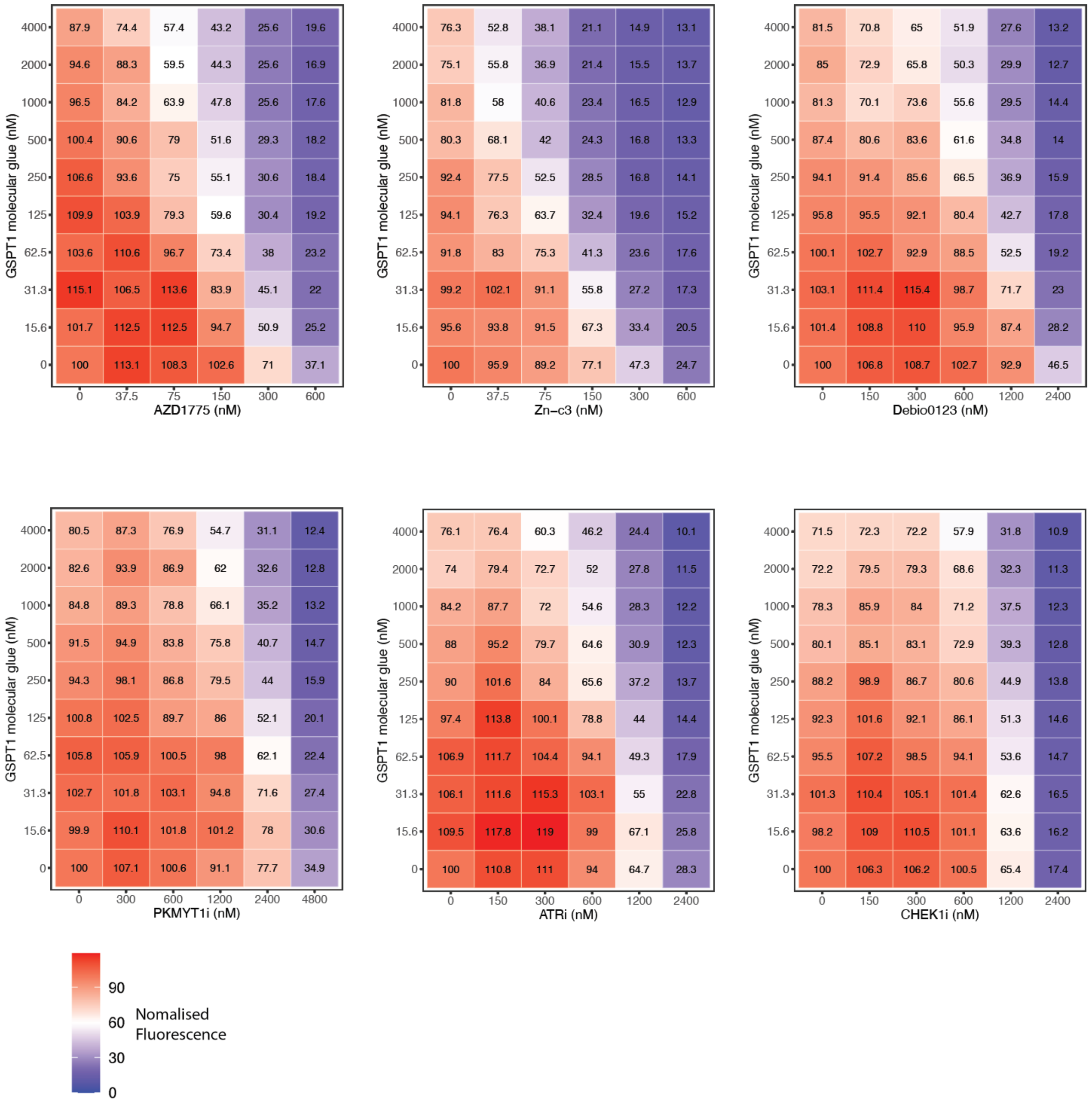

Supplementary Fig.4: Heatmaps of CC-90009-DNA damage response inhibitor combinations. Heatmaps showing resazurin cell viability assays in a 96 well plate format in the RPE TP53$^{-/-}$ cell line. CC-90009 was treated in combination with WEE1 inhibitors (AZD1775, Debio0123 and Zn-c3). PKMYT1i (RP-6306), ATRi (AZD6738) or CHEK1i (LY2603618) for 72 hours (biological n=4). Source data are provided as a Source Data file.

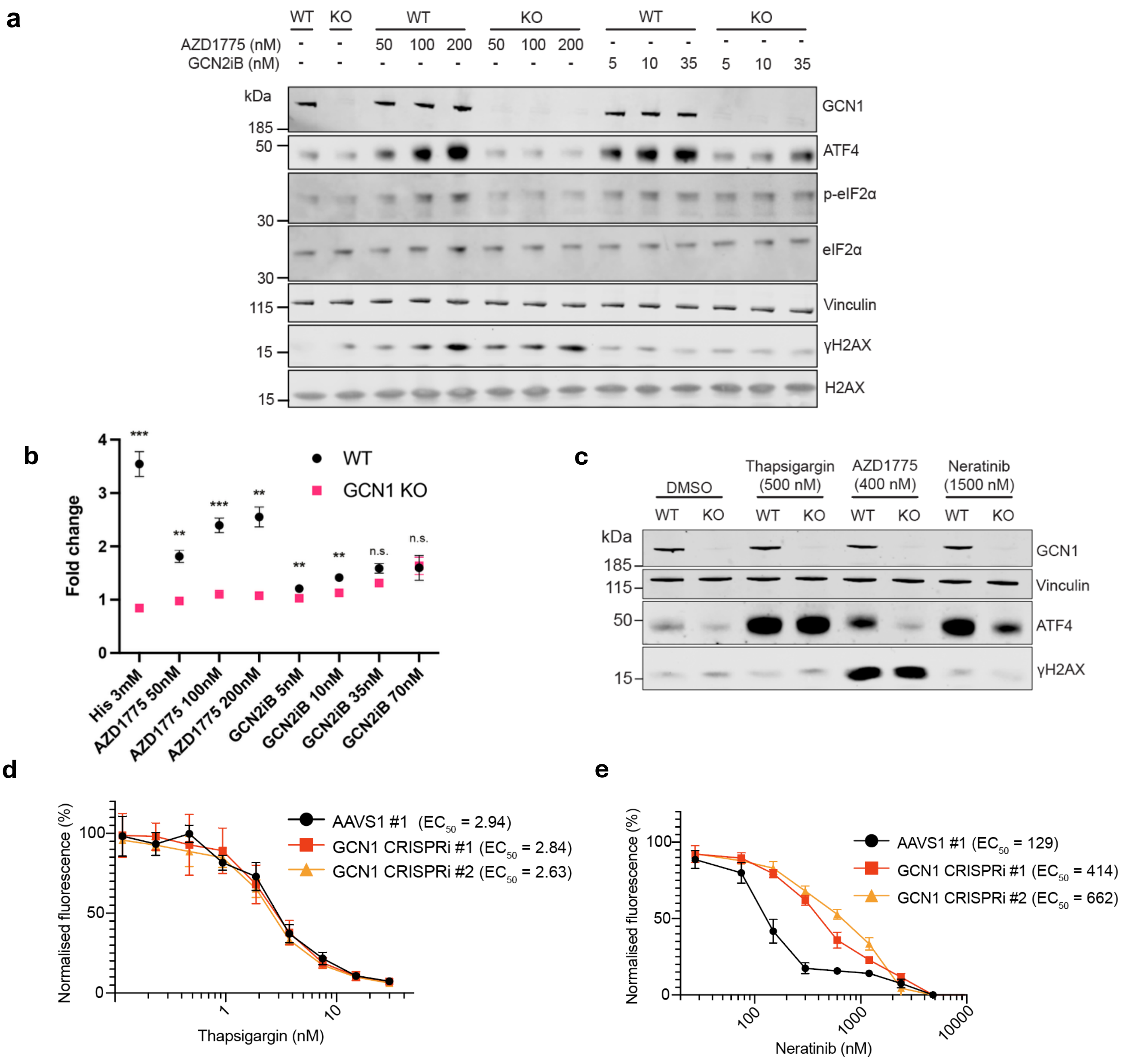

Supplementary Fig.5: Reduced ISR activation from GCN2 activator treatment from GCN1 perturbations a Western blot showing 6 hour treatments of HEK293T wild type and *GCN1*$^{-/-}$ cell lines. Bands of similar molecular weights were run in parallel on separate blots. Total protein (except for ATF4) served as loading controls. **b** A graph showing the fold change of ATF4 reporter[1] signal that was transfected into HEK293T wildtype and *GCN1*$^{-/-}$ cell lines. Transfected cells were treated for 6 hours with either histidinol, AZD1775 or GCN2iB (biological n=3, except for 70 nM GCN2iB which is biological n=2). Graph is depicted with means ± SEM. Statistical analyses comparing WT to *GCN1*$^{-/-}$ was performed using an unpaired two-tailed t-test, n.s. = not significant, ** $p < 0.01$, *** $p < 0.001$. **c** Western blot showing 6 hour treatments of HEK293T wild type and *GCN1*$^{-/-}$ cell lines. **d, e** Resazurin cell viability assay of the RPE TP53$^{-/-}$ dCas9-KRAB cell line expressing either an sgRNA targeting the AAVS1 locus or GCN1 promoter treated with varying concentrations of thapsigargin or neratinib respectively for 72 hours (biological n=4). Graphs are depicted with means ± SD. Source data are provided as a Source Data file.

a

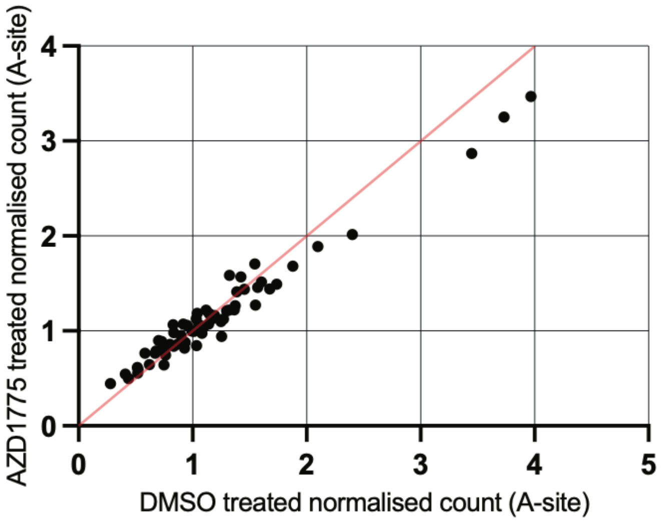

b

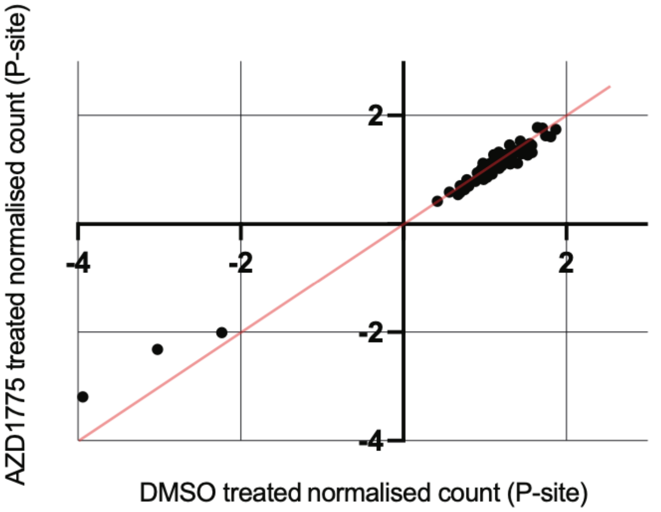

Supplementary Fig.6: A comparison of the ribosome occupancy of AZD1775 treatment vs DMSO. a, b Graphs showing the codon occupancy in the A site and P site of the ribosome respectively. RPE TP53$^{-/-}$ cells were treated with either DMSO or 650 nM for 10 hours. Each point represents a particular codon (biological n=3). Source data are provided as a Source Data file.

a

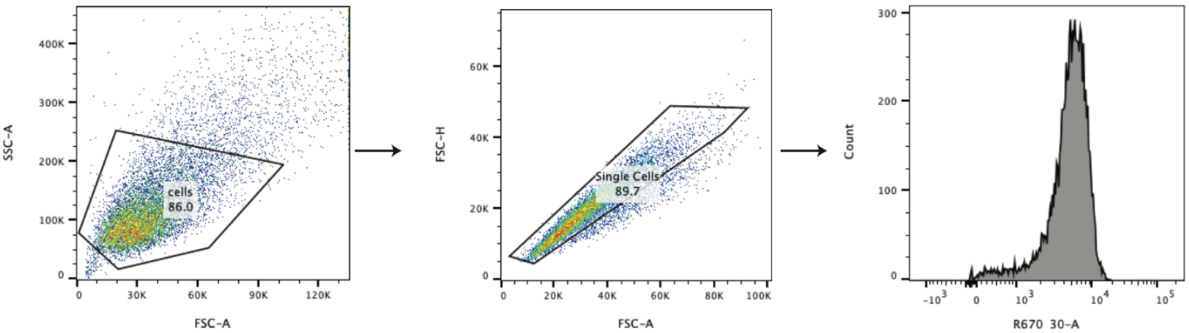

b

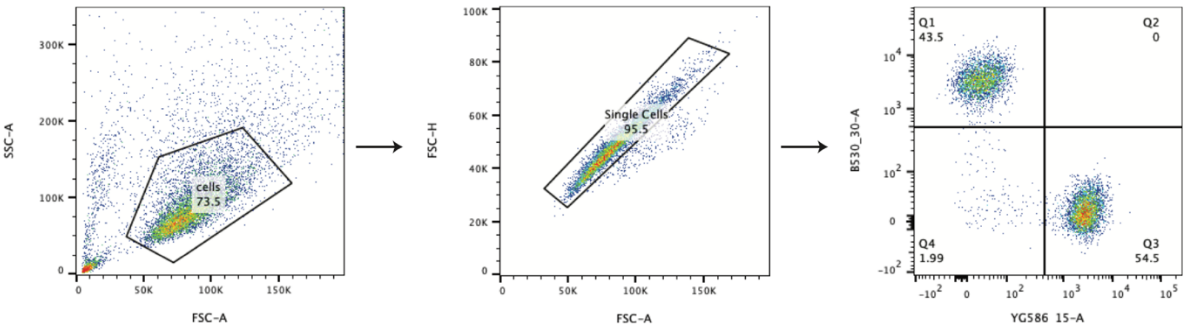

Supplementary Fig.7: Flow cytometry gating strategies. a, b Flow cytometry gating for the AHA click reaction in the RPE TP53$^{-/-}$ cell line and for the CRISPRi-based two colour growth competition assays in the RPE TP53$^{-/-}$ dCas9-KRAB cell line to quantify the GFP: mCherry ratios respectively.

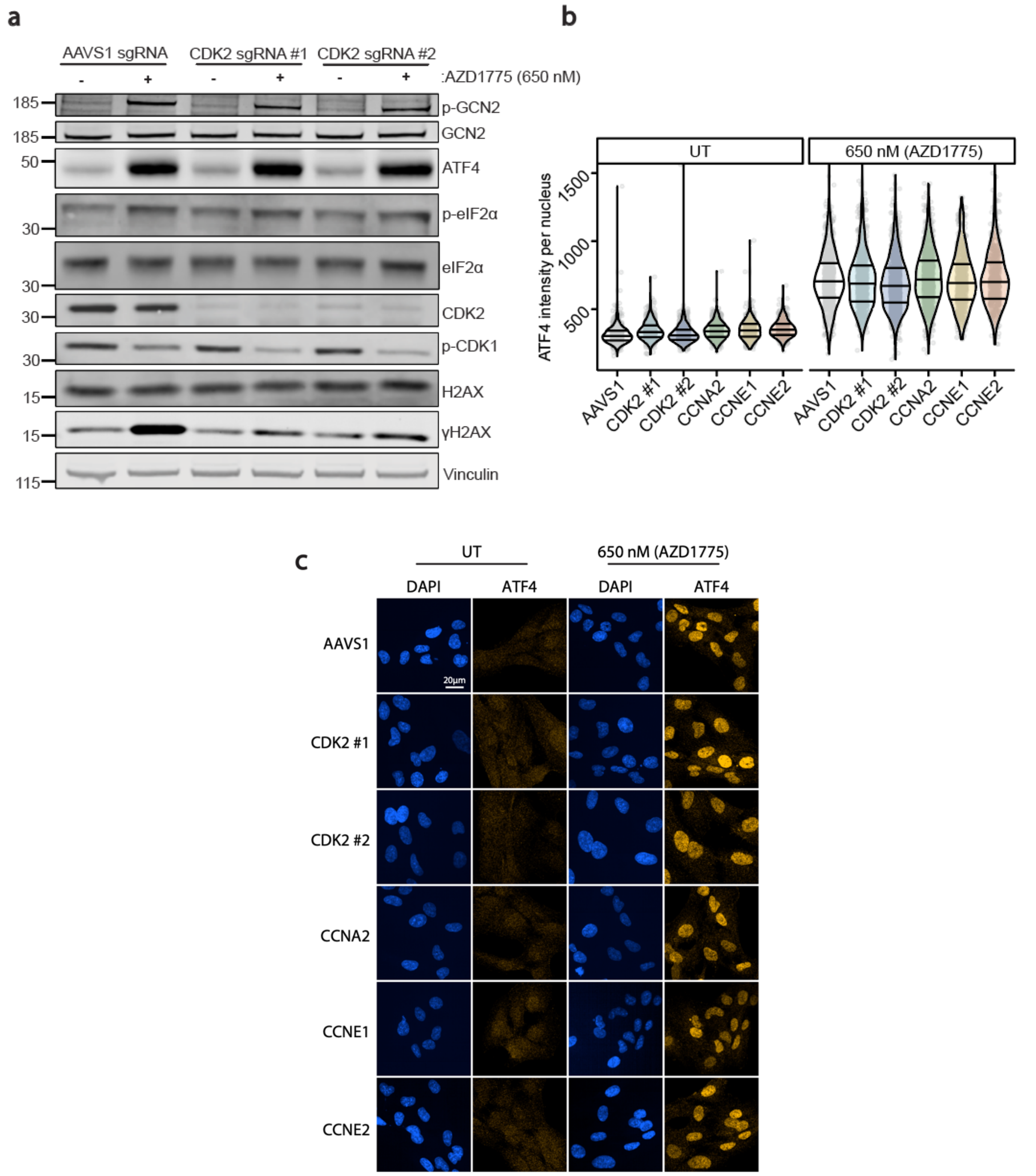

Supplementary Fig.8: The depletion of CDK2, cyclin A2, cyclin E1 or cyclin E2 does not impact WEE1i induced ISR. a Western blot showing the RPE TP53$^{-/-}$ dCas9-KRAB cell line expressing an sgRNA that targets CDK2 or the AAVS1 locus treated with DMSO or 650 nM AZD1775 for 24 hours. Bands of similar molecular weights were run in parallel on separate blots. Total protein (except for ATF4) served as loading controls. Molecular weight markers (kDa) are displayed on the left-hand side. **b** Immunofluorescence probing for nuclear ATF4 in the RPE TP53$^{-/-}$ dCas9-KRAB cell line expressing a sgRNA that targets CDK2 (sgRNA #1 and #2), cyclin A2, cyclin E1 or cyclin E2 or the AAVS1 locus. Cells were treated with DMSO or 650 nM AZD1775 for 24 hours (biological n=3). Violin plots show median and quartile ranges. **c** Representative images of (b). Source data are provided as a Source Data file.

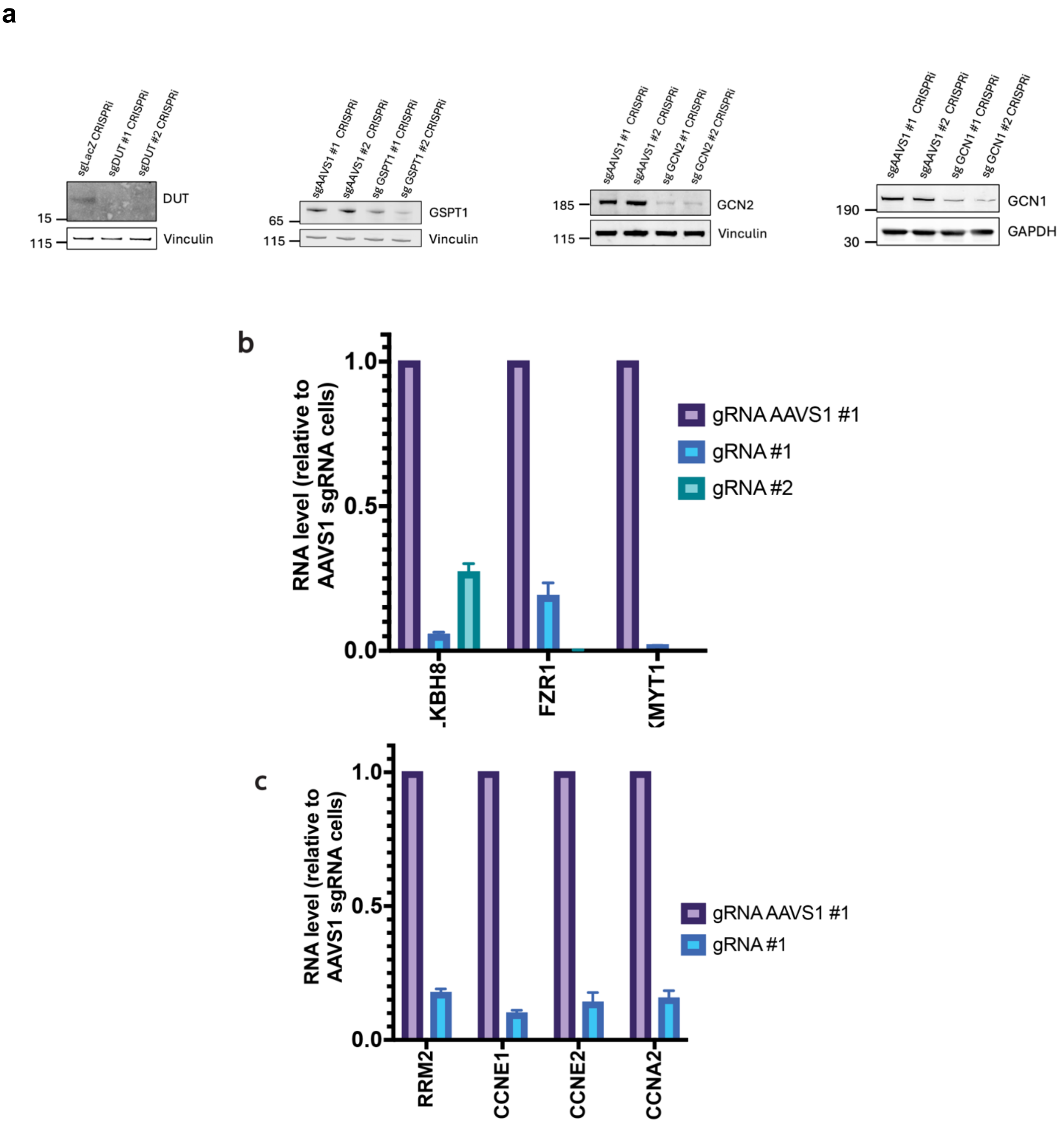

Supplementary Fig.9: Validations for CRISPRi mediated knockdowns in the RPE TP53$^{-/-}$ dCas9-KRAB cell line. a Western blots showing RPE TP53$^{-/-}$ dCas9-KRAB cell lines expressing sgRNAs that target DUT, GSPT1, GCN2 and GCN1 compared to their respective controls that are either non-targeting (LacZ) or targeting the AAVS1 locus. All western blots show molecular weight markers (kDa) on the left-hand side. Western blot validations are representative of biological n=2. **b, c** RT-qPCRs of RPE TP53$^{-/-}$ dCas9-KRAB cell line expressing sgRNAs that target ALKBH8, RRM2, FZR1, PKMYT1, CCNE1, CCNE2 and CCNA2. RNA level was relative to the RPE TP53$^{-/-}$ dCas9-KRAB cell line expressing an sgRNA that target the AAVS1 locus. Measuring the GAPDH RNA abundance for each cell line was used as a control (technical n=3). Graphs are depicted with means ± SD. Source data are provided as a Source Data file.

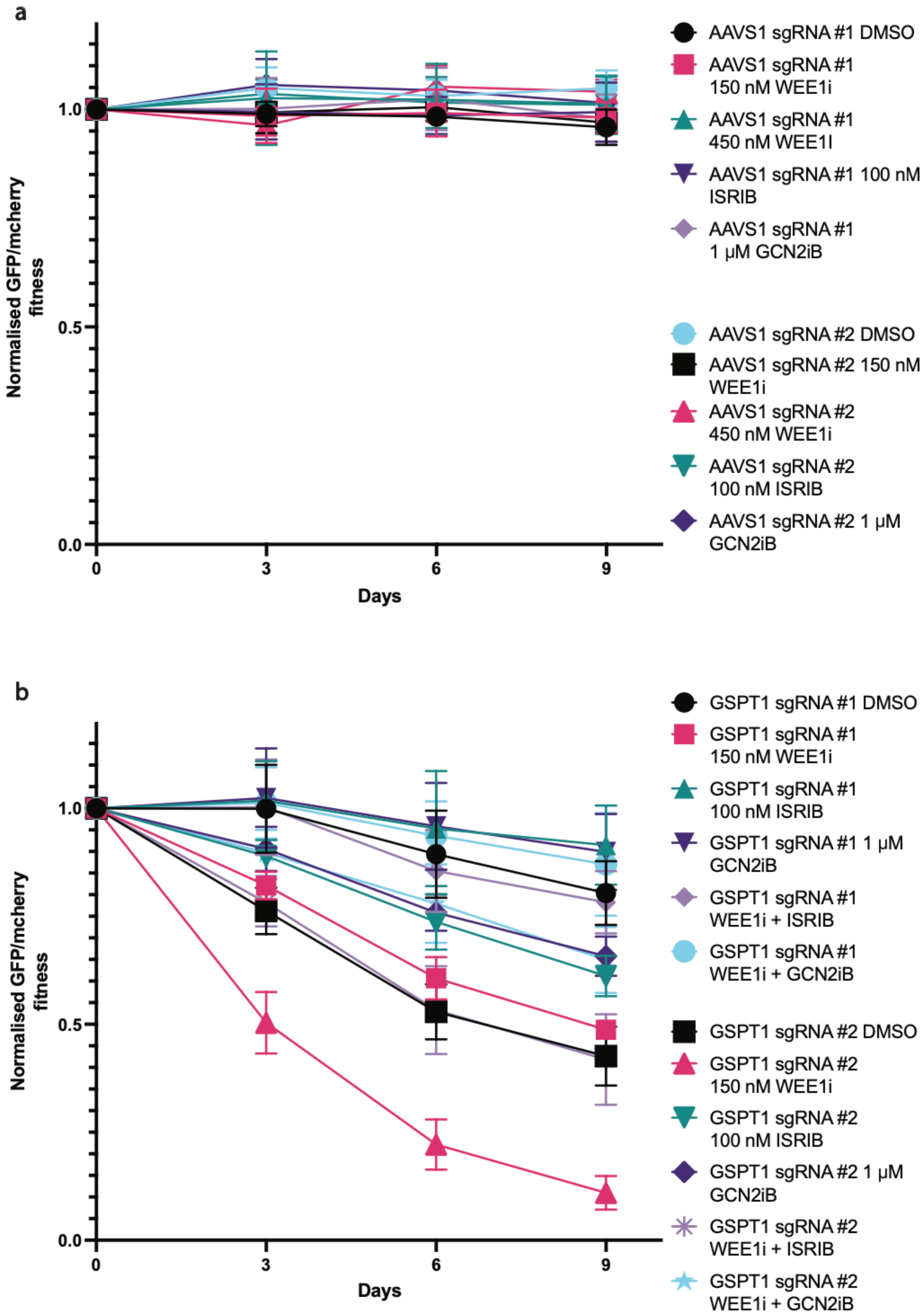
a
Normalised GFP/mcherry fitness
1.0
0.5
0.0
0
3
6
9
Days
AAVS1 sgRNA #1 DMSO
AAVS1 sgRNA #1 150 nM WEE1i
AAVS1 sgRNA #1 450 nM WEE1I
AAVS1 sgRNA #1 100 nM ISRIB
AAVS1 sgRNA #1 1 µM GCN2iB
AAVS1 sgRNA #2 DMSO
AAVS1 sgRNA #2 150 nM WEE1i
AAVS1 sgRNA #2 450 nM WEE1i
AAVS1 sgRNA #2 100 nM ISRIB
AAVS1 sgRNA #2 1 µM GCN2iB
b
Normalised GFP/mcherry fitness
1.0
0.5
0.0
0
3
6
9
Days
GSPT1 sgRNA #1 DMSO
GSPT1 sgRNA #1 150 nM WEE1i
GSPT1 sgRNA #1 100 nM ISRIB
GSPT1 sgRNA #1 1 µM GCN2iB
GSPT1 sgRNA #1 WEE1i + ISRIB
GSPT1 sgRNA #1 WEE1i + GCN2iB
GSPT1 sgRNA #2 DMSO
GSPT1 sgRNA #2 150 nM WEE1i
GSPT1 sgRNA #2 100 nM ISRIB
GSPT1 sgRNA #2 1 µM GCN2iB
GSPT1 sgRNA #2 WEE1i + ISRIB
GSPT1 sgRNA #2 WEE1i + GCN2iB

Supplementary Fig.10: Normalised GFP/mCherry fitness graphs for sgAAVS1-GFP vs sgLacZ-mCherry and sgGSPT1-GFP vs sgLacZ-mCherry. a, b RPE $TP53^{-/-}$ dCas9-KRAB cells expressing sgRNA of interest and GFP were mixed 50/50 with RPE $TP53^{-/-}$ dCas9-KRAB cells expressing sgLacZ-mCherry on day 0. Cells were passaged in a 12 well plate format every 3 days and fresh drug was added. The GFP and mCherry abundance was measured every 3 days for 9 days. Values above 1 indicate the cell population expressing GFP had an increased relative cell fitness compared to cells expressing mCherry; whereas values below 1 indicate the cell population expressing GFP had a reduction in relative cell fitness compared to cells expressing mCherry (biological n=3). Graphs are depicted with means ± SD. Source data are provided as a Source Data file.

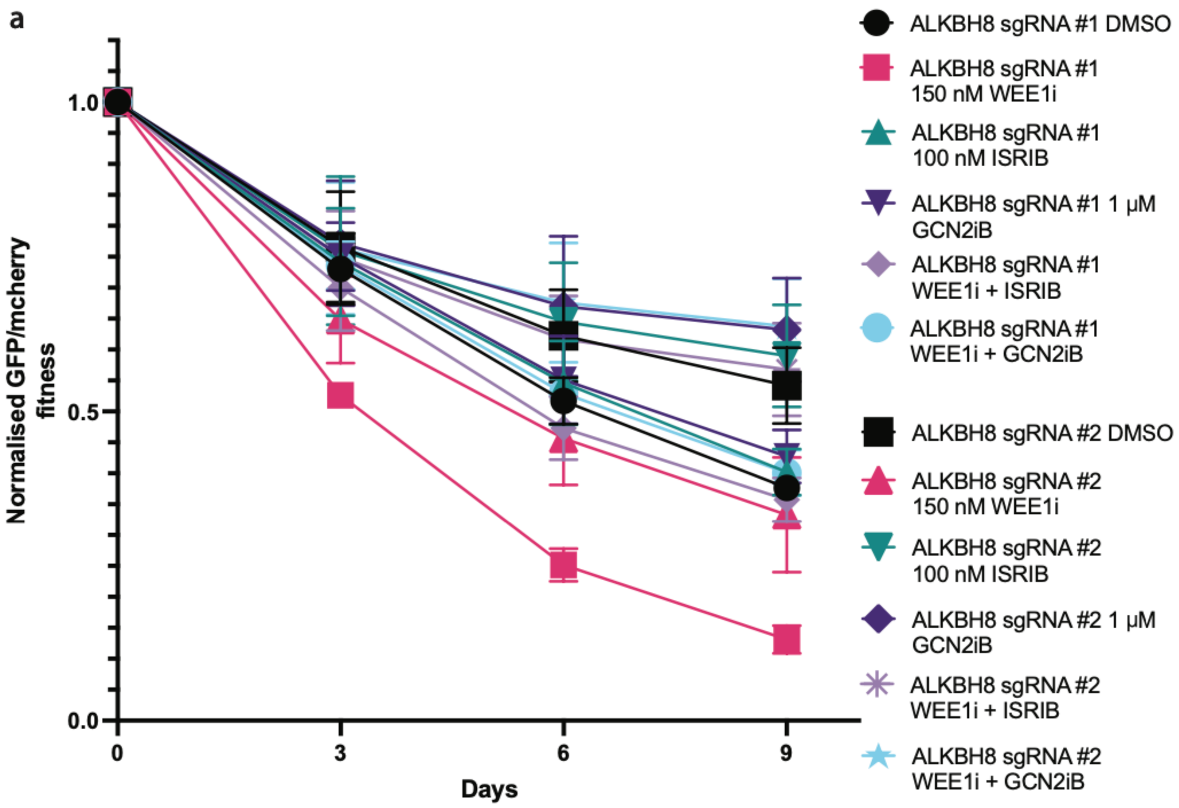

a
Normalised GFP/mcherry fitness
1.0
0.5
0.0
0
3
6
9
Days
ALKBH8 sgRNA #1 DMSO
ALKBH8 sgRNA #1 150 nM WEE1i
ALKBH8 sgRNA #1 100 nM ISRIB
ALKBH8 sgRNA #1 1 μM GCN2iB
ALKBH8 sgRNA #1 WEE1i + ISRIB
ALKBH8 sgRNA #1 WEE1i + GCN2iB
ALKBH8 sgRNA #2 DMSO
ALKBH8 sgRNA #2 150 nM WEE1i
ALKBH8 sgRNA #2 100 nM ISRIB
ALKBH8 sgRNA #2 1 μM GCN2iB
ALKBH8 sgRNA #2 WEE1i + ISRIB
ALKBH8 sgRNA #2 WEE1i + GCN2iB

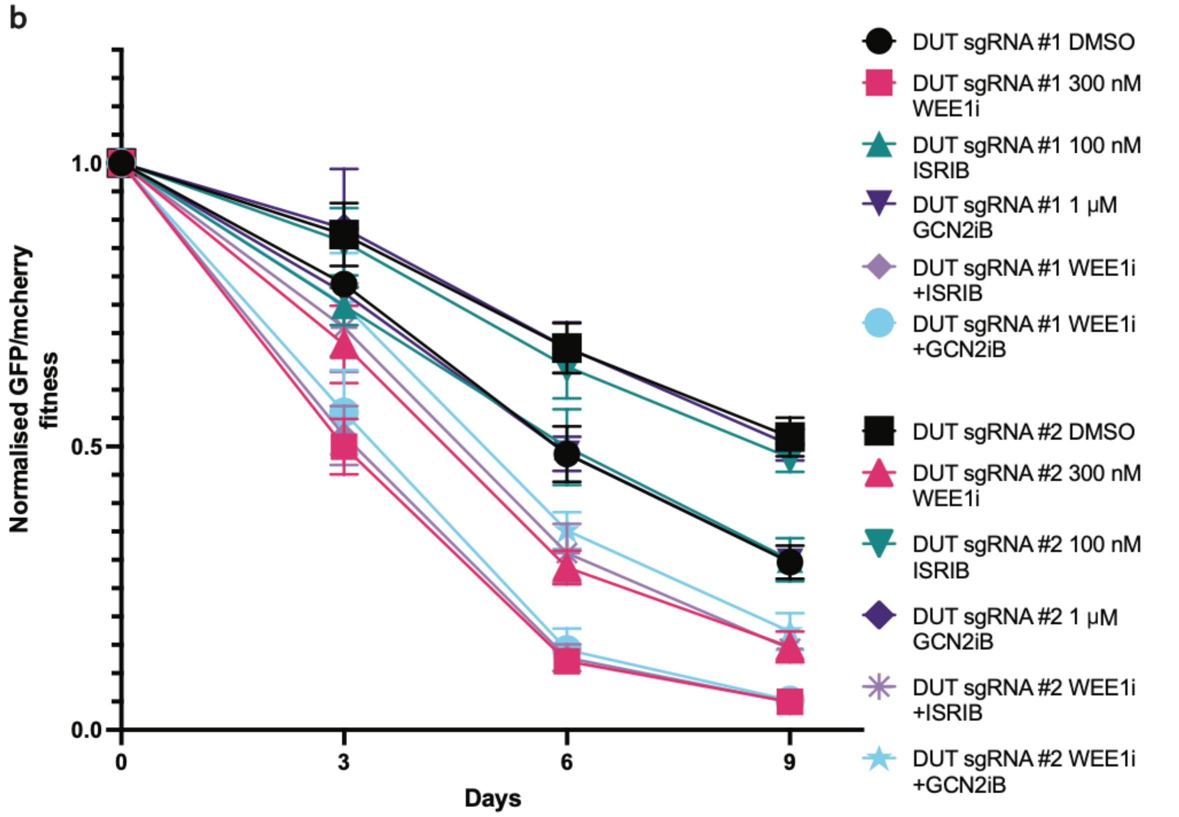

b
Normalised GFP/mcherry fitness
1.0
0.5
0.0
0
3
6
9
Days
DUT sgRNA #1 DMSO
DUT sgRNA #1 300 nM WEE1i
DUT sgRNA #1 100 nM ISRIB
DUT sgRNA #1 1 μM GCN2iB
DUT sgRNA #1 WEE1i +ISRIB
DUT sgRNA #1 WEE1i +GCN2iB
DUT sgRNA #2 DMSO
DUT sgRNA #2 300 nM WEE1i
DUT sgRNA #2 100 nM ISRIB
DUT sgRNA #2 1 μM GCN2iB
DUT sgRNA #2 WEE1i +ISRIB
DUT sgRNA #2 WEE1i +GCN2iB

Supplementary Fig.11: Normalised GFP/mCherry fitness graphs for sgALKBH8-GFP vs sgLacZ-mCherry and sgDUT-GFP vs sgLacZ-mCherry. a, b RPE $TP53^{-/-}$ dCas9-KRAB cells expressing sgRNA of interest and GFP were mixed 50/50 with RPE $TP53^{-/-}$ dCas9-KRAB cells expressing sgLacZ-mCherry on day 0. Cells were passaged in 12 well plate format every 3 days and fresh drug was added. The GFP and mCherry abundance was measured every 3 days for 9 days. Values above 1 indicate the cell population expressing GFP had an increased relative cell fitness compared to cells expressing mCherry; whereas values below 1 indicate the cell population expressing GFP had a reduction in relative cell fitness compared to cells expressing mCherry (biological n=3). Graphs are depicted with means ± SD. Source data are provided as a Source Data file.

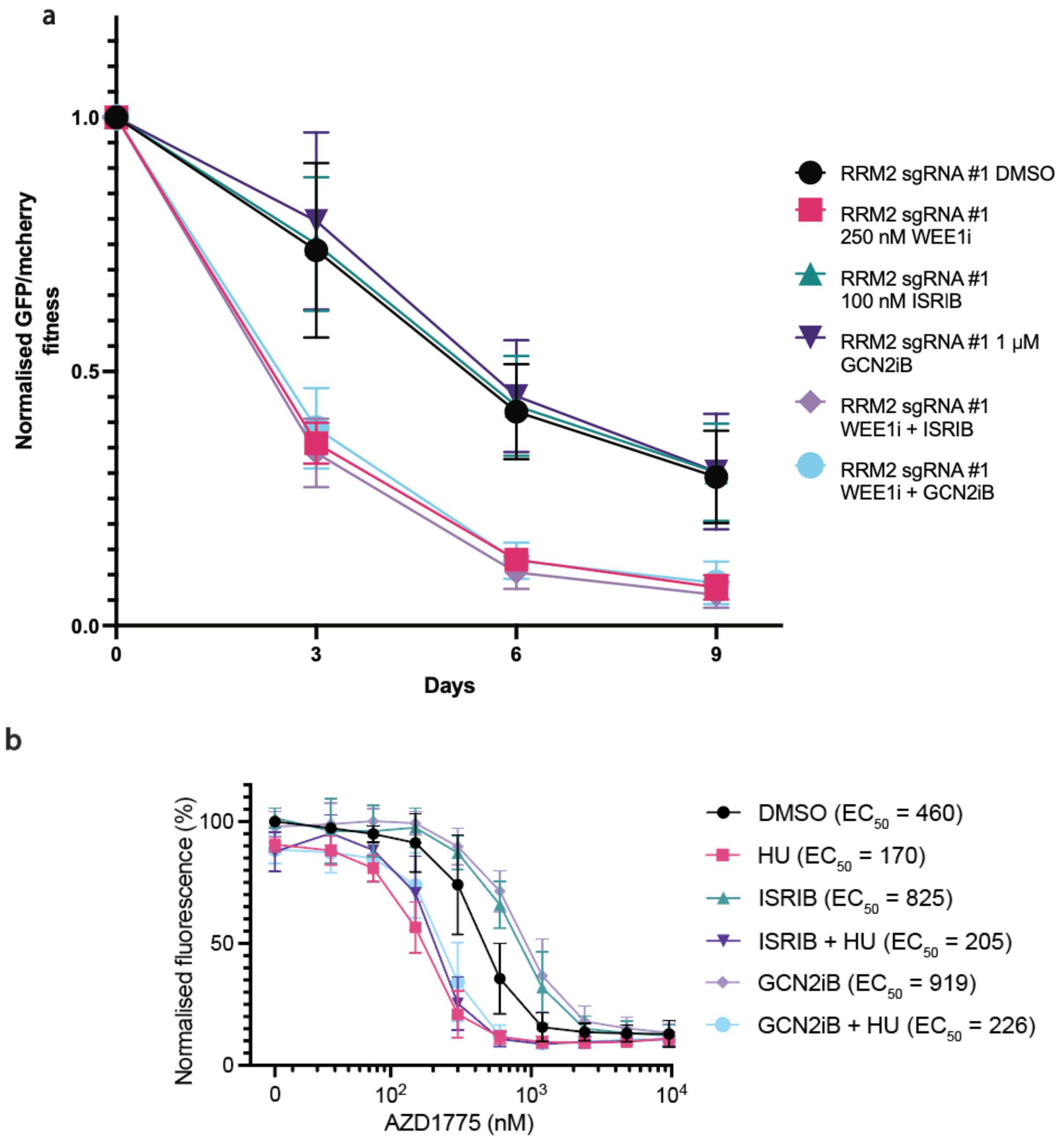

Supplementary Fig.12: Normalised GFP/mCherry fitness graph for sgRRM2-GFP vs sgLacZ-mCherry and cell viability assay of hydroxyurea in combination with AZD1775. a RPE TP53$^{-/-}$ dCas9-KRAB cells expressing sgRRM2 and GFP were mixed 50/50 with RPE TP53$^{-/-}$ dCas9-KRAB cells expressing sgLacZ-mCherry on day 0. Cells were passaged in 12 well plate format every 3 days and fresh drug was added. The GFP and mCherry abundance was measured every 3 days for 9 days. Values above 1 indicate the cell population expressing GFP had an increased relative cell fitness compared to cells expressing mCherry; whereas values below 1 indicate the cell population expressing GFP had a reduction in relative cell fitness compared to cells expressing mCherry (biological n=3). Graphs are depicted with means ± SD. **b** Resazurin cell viability assay with varying concentrations of AZD1775 with and without DMSO, 90 μM hydroxyurea, 100 nM ISRIB, 100 nM ISRIB + 90 μM hydroxyurea, 1 μM GCN1iB and 1 μM GCN1iB + 90 μM hydroxyurea treated on the RPE TP53$^{-/-}$ cell line for 72 hours (biological n=4). Graphs are depicted with means ± SD. Source data are provided as a Source Data file.

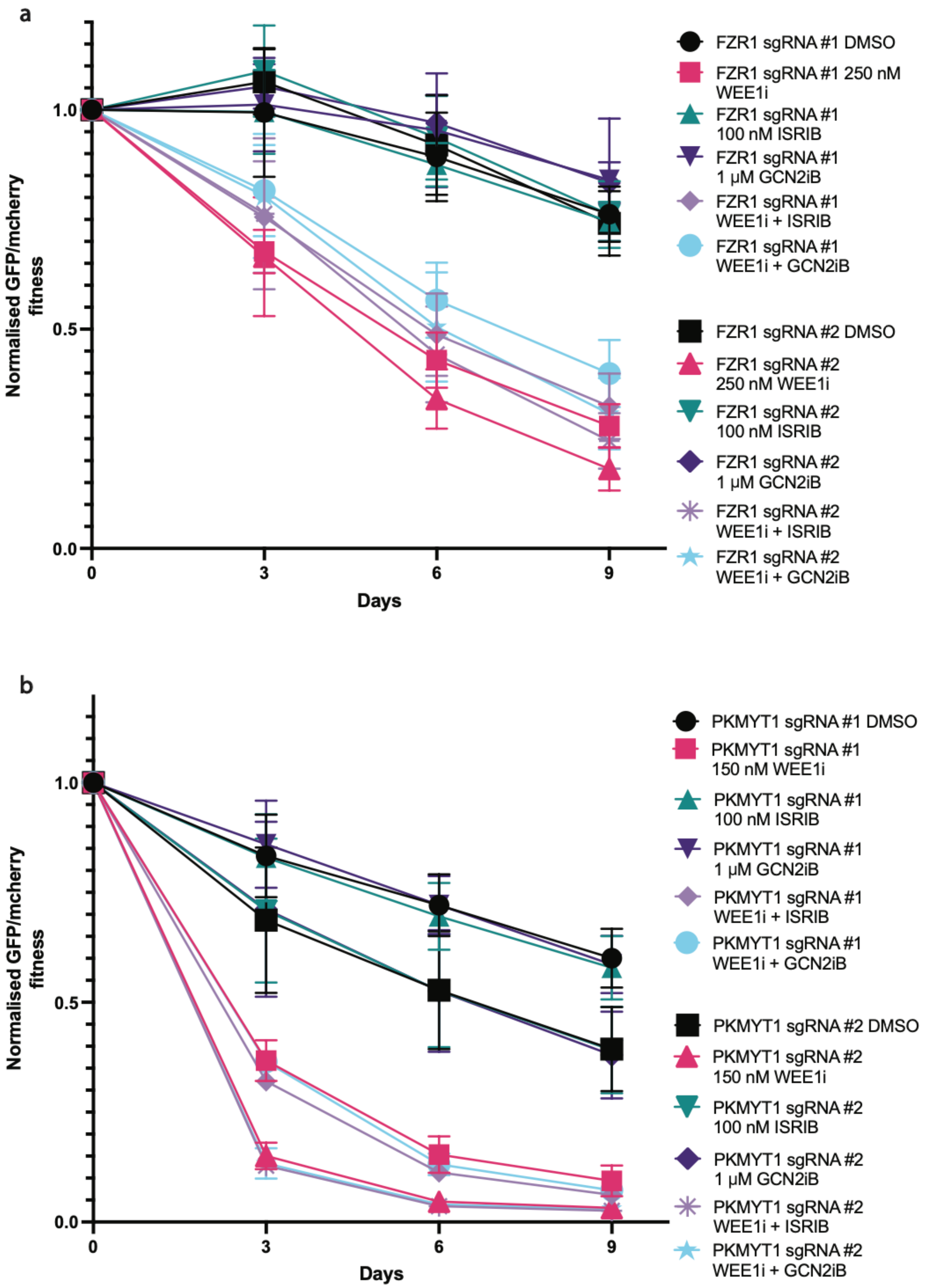
a
Normalised GFP/mcherry fitness
1.0
0.5
0.0
0
3
6
9
Days
FZR1 sgRNA #1 DMSO
FZR1 sgRNA #1 250 nM WEE1i
FZR1 sgRNA #1 100 nM ISRIB
FZR1 sgRNA #1 1 μM GCN2iB
FZR1 sgRNA #1 WEE1i + ISRIB
FZR1 sgRNA #1 WEE1i + GCN2iB
FZR1 sgRNA #2 DMSO
FZR1 sgRNA #2 250 nM WEE1i
FZR1 sgRNA #2 100 nM ISRIB
FZR1 sgRNA #2 1 μM GCN2iB
FZR1 sgRNA #2 WEE1i + ISRIB
FZR1 sgRNA #2 WEE1i + GCN2iB
b
Normalised GFP/mcherry fitness
1.0
0.5
0.0
0
3
6
9
Days
PKMYT1 sgRNA #1 DMSO
PKMYT1 sgRNA #1 150 nM WEE1i
PKMYT1 sgRNA #1 100 nM ISRIB
PKMYT1 sgRNA #1 1 μM GCN2iB
PKMYT1 sgRNA #1 WEE1i + ISRIB
PKMYT1 sgRNA #1 WEE1i + GCN2iB
PKMYT1 sgRNA #2 DMSO
PKMYT1 sgRNA #2 150 nM WEE1i
PKMYT1 sgRNA #2 100 nM ISRIB
PKMYT1 sgRNA #2 1 μM GCN2iB
PKMYT1 sgRNA #2 WEE1i + ISRIB
PKMYT1 sgRNA #2 WEE1i + GCN2iB

Supplementary Fig.13: Normalised GFP/mCherry fitness graphs for sgFZR1-GFP vs sgLacZ-mCherry and sgPKMYT1-GFP vs sgLacZ-mCherry. a, b RPE TP53$^{-/-}$ dCas9-KRAB cells expressing sgRNA of interest and GFP were mixed 50/50 with RPE TP53$^{-/-}$ dCas9-KRAB cells expressing sgLacZ-mCherry on day 0. Cells were passaged in 12 well plate format every 3 days and fresh drug was added. The GFP and mCherry abundance was measured every 3 days for 9 days. Values above 1 indicate the cell population expressing GFP had an increased relative cell fitness compared to cells expressing mCherry; whereas values below 1 indicate the cell population expressing GFP had a reduction in relative cell fitness compared to cells expressing mCherry (biological n=3). Graphs are depicted with means ± SD. Source data are provided as a Source Data file.

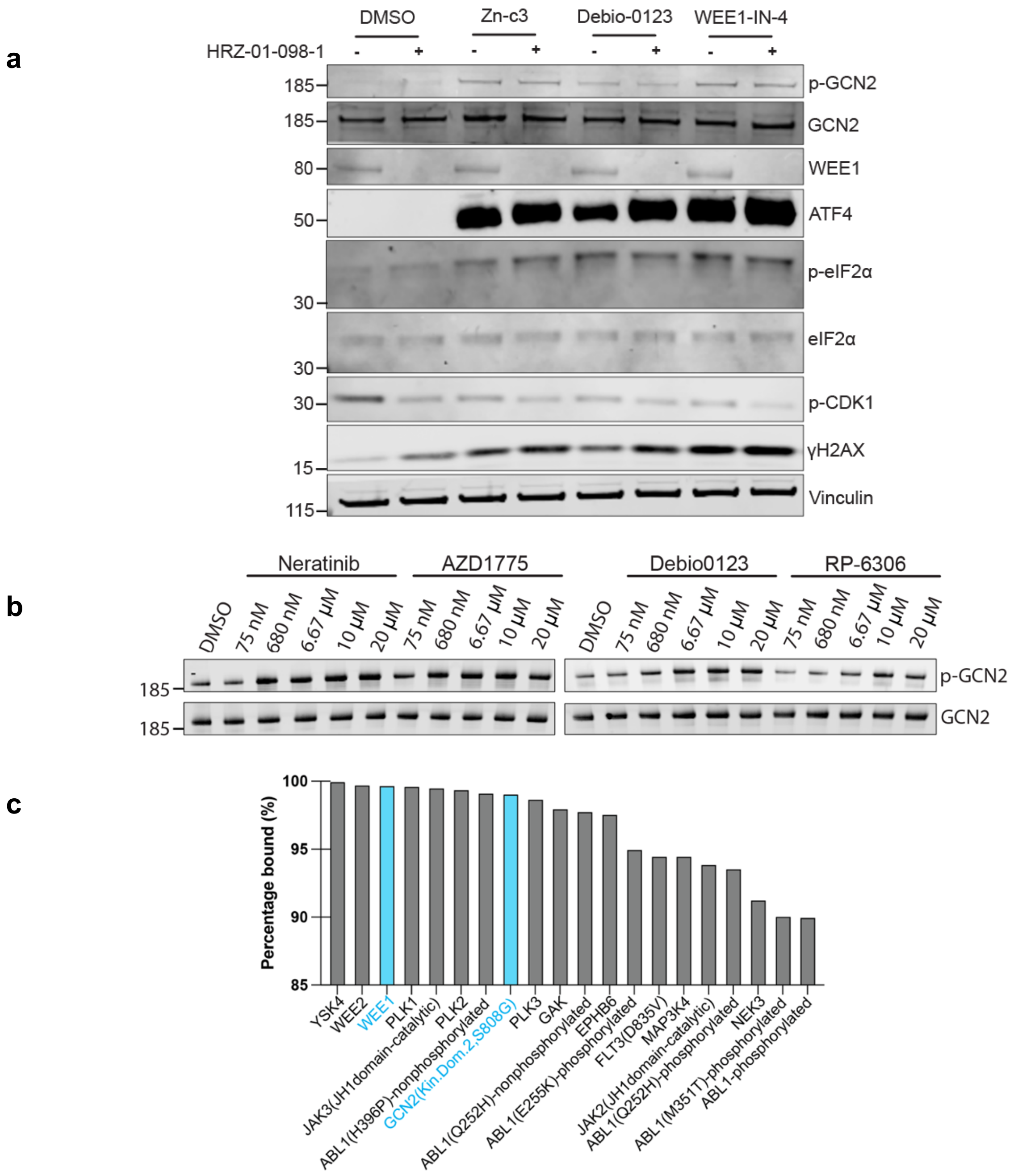

Supplementary Fig.14: WEE1i induced ISR is independent of WEE1. a Western blot of the RPE TP53$^{-/-}$ cell line. Cells were pre-treated with DMSO or 1 μM HRZ-1-098-1 WEE1 molecular glue for 1 hour (7-hour treatment in total) followed by DMSO, 650 nM Zn-c3, 3 μM Debio0123 or 3 μM WEE1-IN-4 for 6 hours. Bands of similar molecular weights were run in parallel on separate blots. Total protein (except for ATF4) served as loading controls. **b** A western blot of an in vitro experiment probing the total and phosphorylated GCN2 in the presence of DMSO, Neratinib, WEE1i (AZD1775 and Debio0123) and PKMYT1i (RP-6306). p-GCN2 and total GCN2 were run in parallel on separate blots. **c** A bar chart showing a rank plot of the top 20 kinases and kinase domains that bound with the 0.5 μM AZD1775 compound from kinome profiling. A total of 403 wild-type and 65 mutant kinases were scanned. Data generated in previous publication[2]. WEE1 and the second domain of GCN2/eIF2AK4 are highlighted in blue. Both western blots shown in this figure display molecular weight markers (kDa) on the left-hand side. Source data are provided as a Source Data file.

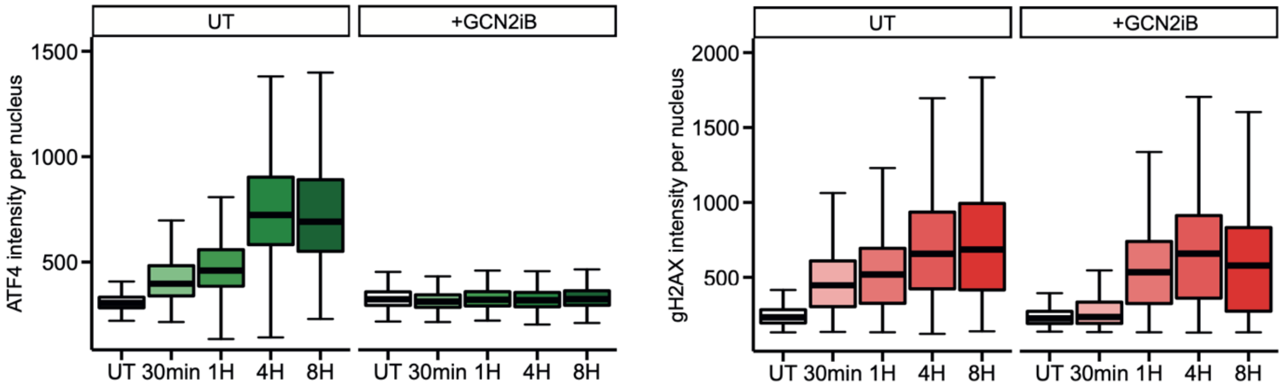

Supplementary Fig.15: ISR induction but not γH2AX from AZD1775 treatment can be rescued by GCN2iB. Immunofluorescence probing for nuclear ATF4 and γH2AX in the RPE $TP53^{-/-}$ cell line. Cells were treated with 650 nM AZD1775 with and without 1 μM GCN2iB at different timepoints (biological n=3). Box plots show the median (centre line), the interquartile range (box), and the minimum and maximum values (whiskers). Source data are provided as a Source Data file.

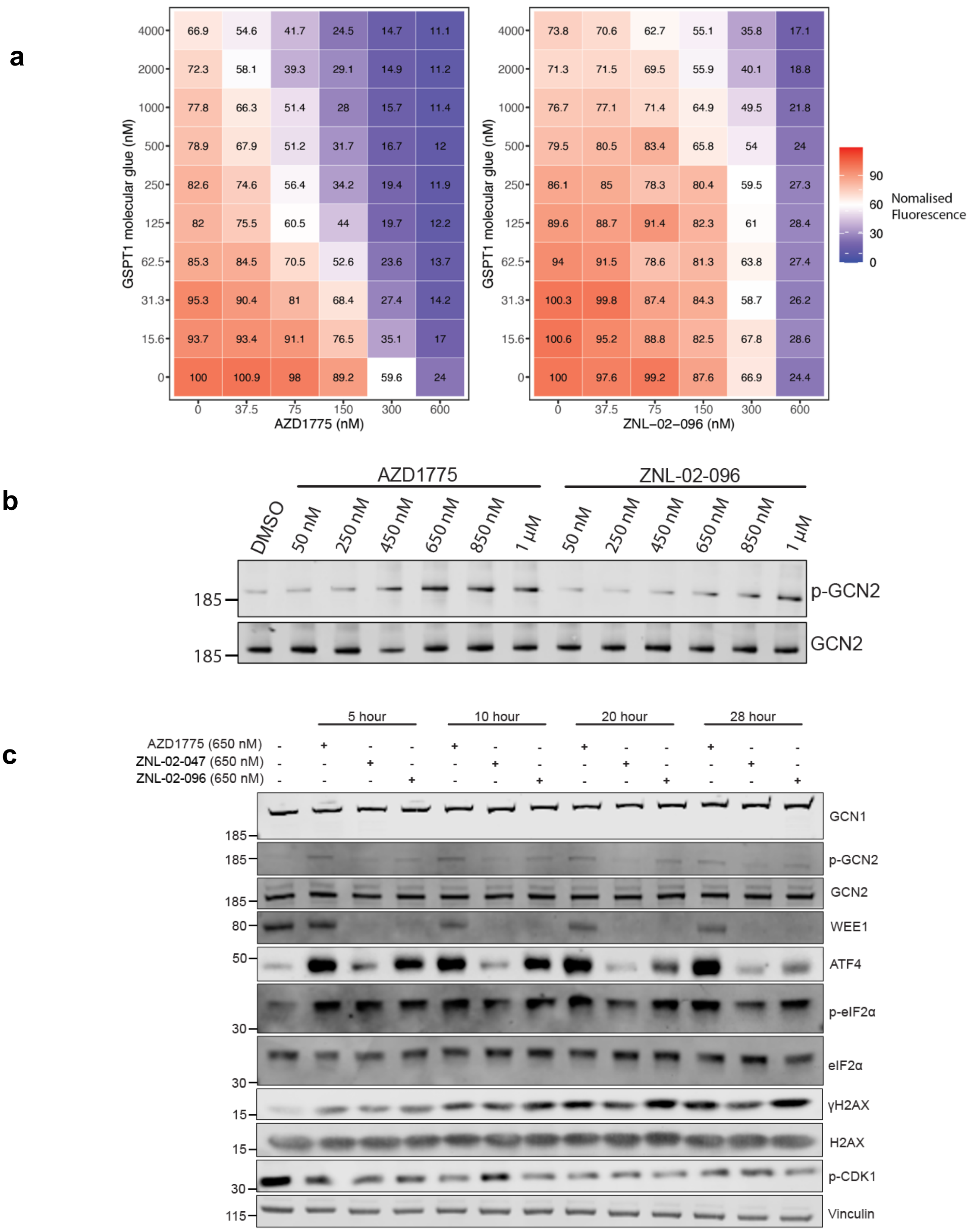

Supplementary Fig.16: A comparison of AZD1775 and PROTAC forms. a Heatmaps showing resazurin cell viability assays in a 96 well plate format. CC-90009 was treated in combination with AZD1775 or ZNL-02-096 for 72 hours (biological n=3). **b** Western blot of an in vitro experiment probing the total and phosphorylated GCN2 in the presence of DMSO, AZD1775 and ZNL-02-096. p-GCN2 and total GCN2 were run in parallel on separate blots. **c** Western blot showing the comparison of 650 nM AZD1775, 650 nM ZNL-02-047 and 650 nM ZNL-02-096 across multiple timepoints for ISR and γH2AX signals in the RPE *TP53*$^{-/-}$ cell line. Bands of similar molecular weights were run in parallel on separate blots. Total protein (except for ATF4) served as loading controls. Both western blots shown in this figure display molecular weight markers (kDa) on the left-hand side. Source data are provided as a Source Data file.

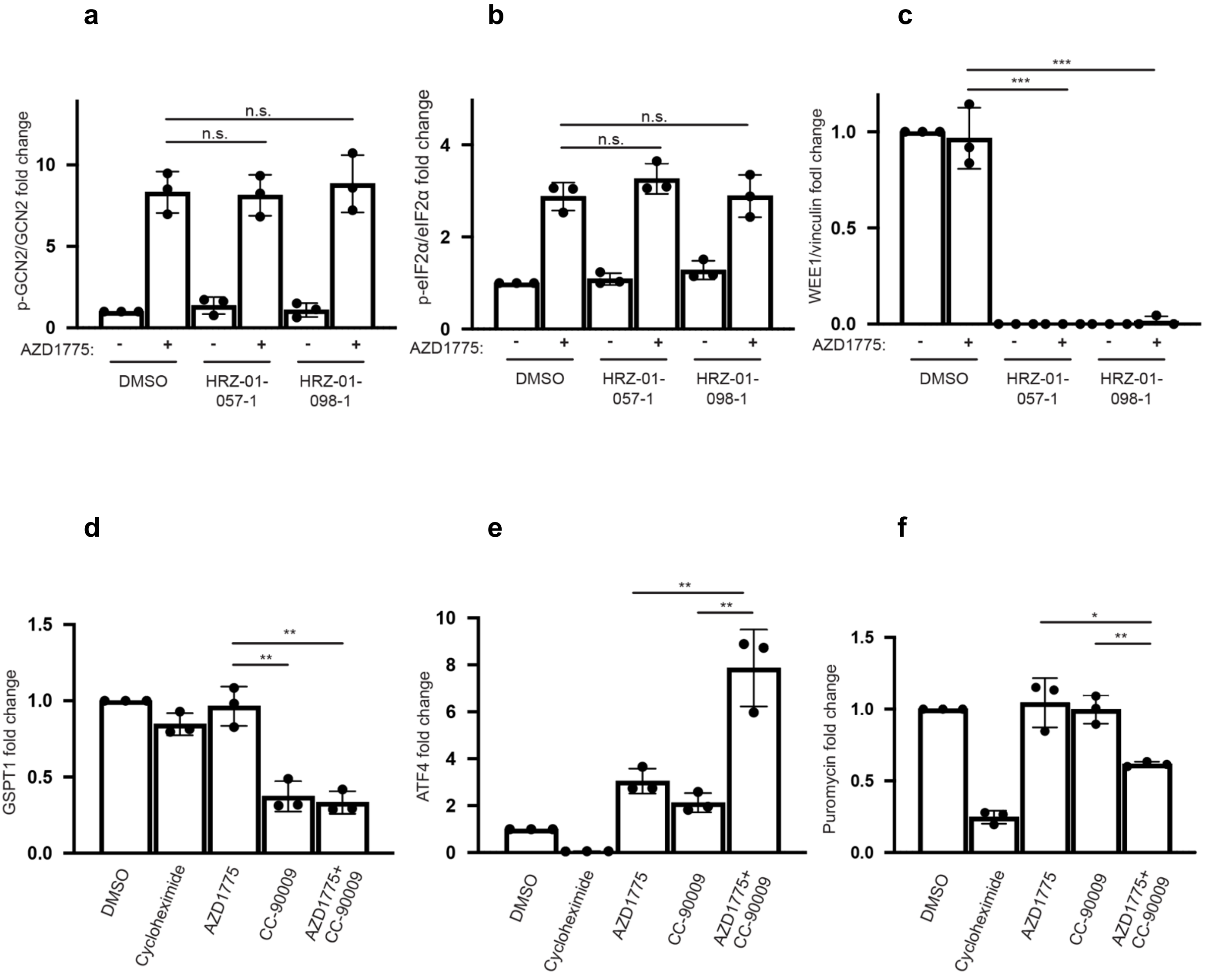

Supplementary Fig.17: Western Blot Quantifications a, b, c Quantifications of western blots performed in Fig.2e. GSPT1 and ATF4 were normalised to vinculin loading control. Puromycin was normalised to ponceau staining (biological n=3). **d, e, f** Quantifications of western blots performed in Fig.4c. p-GCN2 was normalised to total GCN2 total, p-eif2alphα was normalised to total p-eif2alphα and WEE1 was normalised to vinculin loading control (biological n=3). All quantifications were normalised to the vehicle to calculate the fold change of each condition. Bar charts are depicted with means ± SD. Statistical analyses were performed using unpaired two-tailed t-tests, n.s. = not significant, * $p < 0.05$, ** $p < 0.01$, *** $p < 0.001$. Source data are provided as a Source Data file.

Supplementary Table 1. List of antibodies used in this study

Target	Supplier	Catalogue no.	Application	Dilution	Species
WEE1	Cell signaling	CST4936	Western	1:500	Rabbit
CDK1	Abcam	ab32094	Western	1:500	Rabbit
CDK1 pY15	Cell signaling	CST9111	Western	1:500	Rabbit
H2AX	Abcam	ab11175	Western	1:500	Rabbit
gH2AX pS139	Merck	05-636	Western	1:1000	Mouse
Vinculin	Abcam	ab219649	Western	1:3000	Rabbit
GAPDH	Merck	MAB374	Western	1:5000	Mouse
GCN2	Abcam	ab134053	Western	1:500	Rabbit
GCN2 pT899	Abcam	ab75836	Western	1:500	Rabbit
eIF2α	Cell signaling	CST9722	Western	1:500	Rabbit
eIF2α pS51	Cell signaling	CST3597	Western	1:500	Rabbit
ATF4	Cell signaling	CST11815	Western	1:500	Rabbit
GSPT1	Abcam	ab49878	Western	1:500	Rabbit
DUT	Proteintech	13740-1-AP	Western	1:500	Rabbit
GCN1	Abcam	ab86139	Western	1:500	Rabbit
CDK2	Cell signaling	CST2546	Western	1:500	Rabbit
Puromycin	Merck	MABE343	Western	1:2000	Mouse
gH2AX pS139	Merck	05-636	IF	1:1000	Mouse
ATF4	Cell signaling	CST11815	IF	1:200	Rabbit

Supplementary Table 2. List of sgRNAs and primers used in the study

Target	sgRNA sequence (5'-3')	FW primer (5'-3')	RV primer (5'-3')
sgRNAs used for CRISPRi mediated knockdown			
GCN2 (#1)	GCAGCGCTGCGCCCAAGGCA		
GCN2 (#2)	GGCCCACCGCCGCCCAGGCA		
GCN1 (#1)	GGGCGGCGCAGGCAGACCGC		
GCN1 (#2)	GGGCGGACACGCAGGTGAGG		
GSPT1 (#1)	GAGCTAGCGACAAAGATCCC		
GSPT1 (#2)	GCTCGCGACGACGACAGAGG		
ALKBH8 (#1)	GGGCGTGCAAGTATCCGCTG		
ALKBH8 (#2)	GTGGCCGCGCCCAGGGGAGA		
DUT (#1)	GCGAGCGAGGAGACCACCGG		
DUT (#2)	GAGGCGAGCGAGGAGACCAC		
RRM2 (#1)	GGGACAGGACGGCTGGGACA		
FZR1 (#1)	GGCGGTCCCTAATATGGCGG		
FZR1 (#2)	GTCCGCGGTCCCTAATATGG		
PKMYT1 (#1)	GTCACGGGAGTCCTCCGCCC		
PKMYT1 (#2)	GGGGCGTCCGGAACAGTCGA		
CDK2 (#1)	GCCGTGGCCCCGGGTCGGGA		
CDK2 (#2)	GTGGCGGTCGGGAACTCGGT		
CCNE1 (#1)	GGCCGCCAGCGCGGTGTAGG		
CCNE2 (#1)	GCCGATCACTTACCACAGGC		
CCNA2 (#1)	GACTGAAGTCCGGGAACCCG		
AAVS1 (#1)	ACTGTTGACGGCGGCGATGT		
AAVS1 (#2)	GCTGATACCGTCGGCGTTGG		
Primers used for RT-qPCR			
GAPDH		GTGGTCTCCTCTGACTTCAAC	GGAAATGAGCTTGACAAAGTGG
ALKBH8		TGAGGCAAACACCTTGTAACT	CAGCCGTGAGGCTTCTTTAT
RRM2		GCAAGCGATGGCATAGTAAATG	AACAGCGGGCTTCTGTAATC
FZR1		CTGAGGTTCTGGAACGTCTTTAG	TACCGGATCCTGGTGAAGAG
PKMYT1		AGTGGCATGCAACATGGAG	AGCATCATGACAAGGACAGAAC
CCNE1		GTGACAGATGGAGCTTGTTC	CATTCAGCCAGGACACAATAG
CCNE2		AGTCCAGTGAAGCTGAAGAC	TCCTCCAGCATAGCCAAATAG
CCNA2		GATAGGTTCCTGTCTTCCATGT	TACACAAACTCTGCTACTTCTGG

References

1. Emanuelli, G., Zhu, J., Li, W., Morrell, N. W. & Marciniak, S. J. Functional validation of *EIF2AK4* (GCN2) missense variants associated with pulmonary arterial hypertension. *Hum. Mol. Genet.* ddae082 (2024) doi:10.1093/hmg/ddae082.
2. Zhu, J.-Y. *et al.* Structural Basis of Wee Kinases Functionality and Inactivation by Diverse Small Molecule Inhibitors. *J. Med. Chem.* **60**, 7863–7875 (2017).
