# Supplementary material for: WEE1 inhibitors synergise with mRNA translation defects via activation of the kinase GCN2
Source: Nat Commun. 2025 Oct 9;16:8983. doi: 10.1038/s41467-025-64050-5 (PMC12511557; doi:10.1038/s41467-025-64050-5)
Supplement: Supplementary file 2 — Description of Additional Supplementary Files [file 41467_2025_64050_MOESM2_ESM.pdf]

Description of Additional Supplementary Files

File Name: Supplementary Data 1

Description: The sgRNA sequences of the CRISPRi library
