# Supplementary material for: WEE1 inhibitors synergise with mRNA translation defects via activation of the kinase GCN2
Source: Nat Commun. 2025 Oct 9;16:8983. doi: 10.1038/s41467-025-64050-5 (PMC12511557; doi:10.1038/s41467-025-64050-5)
Supplement: Supplementary file 4 — Reporting Summary [file 41467_2025_64050_MOESM4_ESM.pdf]

nature portfolio

Corresponding author(s): Stephen P. Jackson

Last updated by author(s): 2025/08/24

Reporting Summary

Nature Portfolio wishes to improve the reproducibility of the work that we publish. This form provides structure for consistency and transparency in reporting. For further information on Nature Portfolio policies, see our Editorial Policies and the Editorial Policy Checklist.

Statistics

For all statistical analyses, confirm that the following items are present in the figure legend, table legend, main text, or Methods section.

n/a	Confirmed	
☐	☒	The exact sample size (*n*) for each experimental group/condition, given as a discrete number and unit of measurement
☐	☒	A statement on whether measurements were taken from distinct samples or whether the same sample was measured repeatedly
☐	☒	The statistical test(s) used AND whether they are one- or two-sided<br>*Only common tests should be described solely by name; describe more complex techniques in the Methods section.*
☐	☒	A description of all covariates tested
☐	☒	A description of any assumptions or corrections, such as tests of normality and adjustment for multiple comparisons
☐	☒	A full description of the statistical parameters including central tendency (e.g. means) or other basic estimates (e.g. regression coefficient) AND variation (e.g. standard deviation) or associated estimates of uncertainty (e.g. confidence intervals)
☐	☒	For null hypothesis testing, the test statistic (e.g. *F*, *t*, *r*) with confidence intervals, effect sizes, degrees of freedom and *P* value noted<br>*Give P values as exact values whenever suitable.*
☒	☐	For Bayesian analysis, information on the choice of priors and Markov chain Monte Carlo settings
☒	☐	For hierarchical and complex designs, identification of the appropriate level for tests and full reporting of outcomes
☒	☐	Estimates of effect sizes (e.g. Cohen's *d*, Pearson's *r*), indicating how they were calculated

Our web collection on statistics for biologists contains articles on many of the points above.

Software and code

Policy information about availability of computer code

Data collection	All specific softwares used for data collection are detailed in material and methods section.
Data analysis	GraphPad Prism 10, ImageJ 1.53t, FlowJo v.10.8.1, Revvity Harmony v5.2 and microsoft excel were used in the analysis of data in this study.

For manuscripts utilizing custom algorithms or software that are central to the research but not yet described in published literature, software must be made available to editors and reviewers. We strongly encourage code deposition in a community repository (e.g. GitHub). See the Nature Portfolio guidelines for submitting code & software for further information.

Data

Policy information about availability of data

All manuscripts must include a data availability statement. This statement should provide the following information, where applicable:

- Accession codes, unique identifiers, or web links for publicly available datasets
- A description of any restrictions on data availability
- For clinical datasets or third party data, please ensure that the statement adheres to our policy

All data is appropriately submitted as supplemental files. CRISPRi and ribo-seq data have accession codes on ENA/European Nucleotide Archive, these sequencing data are publicly available

Research involving human participants, their data, or biological material

Policy information about studies with human participants or human data. See also policy information about sex, gender (identity/presentation), and sexual orientation and race, ethnicity and racism.

Reporting on sex and gender	N/A
Reporting on race, ethnicity, or other socially relevant groupings	N/A
Population characteristics	N/A
Recruitment	N/A
Ethics oversight	N/A

Note that full information on the approval of the study protocol must also be provided in the manuscript.

Field-specific reporting

Please select the one below that is the best fit for your research. If you are not sure, read the appropriate sections before making your selection.

☒ Life sciences ☐ Behavioural & social sciences ☐ Ecological, evolutionary & environmental sciences

For a reference copy of the document with all sections, see nature.com/documents/nr-reporting-summary-flat.pdf

Life sciences study design

All studies must disclose on these points even when the disclosure is negative.

Sample size	CRISPR screen was performed with 2 separate biological replicates. Sample size/sgRNA representation in CRISPR screen indicated in the methods section. ISR phenotype observed across multiple WEE1 inhibitors (AZD1775, Zn-c3, Debio0123, WEE1-IN-4). AZD1775 induced ISR was observed in non-cancer, human, mouse cell lines as well as patient derived organoid material.
Data exclusions	No data exclusions were performed in this study
Replication	WEE1i induced ISR was observed in quadruplicate across multiple cancer, non-cancer, human, mouse cell lines. The number of replicates performed is indicated in the respective figure legends. All statistics performed were on experiments performed either in triplicate or quadruplicate. All resazurin viability assays to calculate EC50 concentrations were performed at a minimum of 3 biological replicates. All immunofluorescence experiments were performed at a minimum of 3 biological replicates. CRISPR screen was performed with 2 separate biological replicates. Ribosome profiling was perfomed on 3 biological replicates.
Randomization	For microscopy-based analysis, a fixed number of images were obtained randomly from each sample and analysed together with control samples using the same analysis pipeline. For cell culture and biochemical assays, sample conditions for an experiment often came from the same source (eg same cell line, same buffer preparation, same stock of media etc) making variation low thereby making randomisation less crucial.
Blinding	No blinding was used during sample collection and processing. However, unless indicated, image acquisition and data analyses were performed in an unbiased way using high-throughput high-content Opera Phenix microscope and Harmony software. Samples for immunoblotting or samples that required the addition of different genotoxic compounds were known to the experimenter when preparing samples. The outcome of such experiments were often objective, so the risk of unconscious bias in perparation is low. Many of our experiment readouts were also quantitative machine-based readouts thereby making bias risk even lower.

Reporting for specific materials, systems and methods

We require information from authors about some types of materials, experimental systems and methods used in many studies. Here, indicate whether each material, system or method listed is relevant to your study. If you are not sure if a list item applies to your research, read the appropriate section before selecting a response.

Materials & experimental systems

n/a	Involved in the study	
☐	☒	Antibodies
☐	☒	Eukaryotic cell lines
☒	☐	Palaeontology and archaeology
☒	☐	Animals and other organisms
☒	☐	Clinical data
☒	☐	Dual use research of concern
☒	☐	Plants

Methods

n/a	Involved in the study	
☒	☐	ChIP-seq
☐	☒	Flow cytometry
☒	☐	MRI-based neuroimaging

Antibodies

Antibodies used

Target, Supplier, Catalogue no., Application, Dilution, Species
WEE1, Cell signaling, CST4936, Western, 1:500, Rabbit
CDK1, Abcam, ab32094, Western, 1:500, Rabbit
CDK1 pY15, Cell signaling, CST9111, Western, 1:500, Rabbit
H2AX, Abcam, ab11175, Western, 1:500, Rabbit
gH2AX pS139, Merck, 05-636, Western, 1:1000, Mouse
Vinculin, Abcam, ab219649, Western, 1:3000, Rabbit
GAPDH, Merck, MAB374, Western, 1:5000, Mouse
GCN2, Abcam, ab134053, Western, 1:500, Rabbit
GCN2 pT899, Abcam, ab75836, Western, 1:500, Rabbit
eIF2α, Cell signaling, CST9722, Western, 1:500, Rabbit
eIF2α pS51, Cell signaling, CST3597, Western, 1:500, Rabbit
ATF4, Cell signaling, CST11815, Western, 1:500, Rabbit
GSPT1, Abcam, ab49878, Western, 1:500, Rabbit
DUT, Proteintech, 13740-1-AP, Western, 1:500, Rabbit
GCN1, Abcam, ab86139, Western, 1:500, Rabbit
CDK2, Cell signaling, CST2546, Western, 1:500, Rabbit
Puromycin, Merck, MABE343, Western, 1:2000, Mouse
gH2AX pS139, Merck, 05-636, IF, 1:1000, Mouse
ATF4, Cell signaling, CST11815, IF, 1:200, Rabbit
anti-rabbit secondary, LI-COR 926-68021, Western, 1:10,000, Goat
anti-mouse secondary, LI-COR, 926-32212, Western, 1:10,000, Goat
HRP-conjugated secondary antibody, Santa-Cruz, sc-2313, Western, 1:10,000, Goat

Validation

Most antibodies were validated by the positive control conditions included in each experiment. Commercially available antibodies were also validated by the supplier. Please see more details in the manufacturer's websites for further details.

Eukaryotic cell lines

Policy information about cell lines and Sex and Gender in Research

Cell line source(s)

RPE TP53-/- dCas9 KRAB cell line provided by the Corn Lab.
GCN1 KO HEK293T and HEK293T WT were provided by Abcam (ab255449 and ab266780)
All other cell lines were obtained from ATCC.

Authentication

Cell lines were not authenticated. All knockout and CRISPRi depletions were validated by western blot or RT-qPCR.

Mycoplasma contamination

All cell lines were mycoplasma tested prior to storage in liquid nitrogen and this was carried out each time any vials were entered into liquid nitrogen. No samples tested positive throughout the duration of these experiments.

Commonly misidentified lines
(See ICLAC register)

No commonly misidentified cell lines were used in this study

Plants

Seed stocks	N/A
Novel plant genotypes	N/A
Authentication	N/A

Flow Cytometry

Plots

Confirm that:

☒ The axis labels state the marker and fluorochrome used (e.g. CD4-FITC).

☒ The axis scales are clearly visible. Include numbers along axes only for bottom left plot of group (a 'group' is an analysis of identical markers).

☒ All plots are contour plots with outliers or pseudocolor plots.

☒ A numerical value for number of cells or percentage (with statistics) is provided.

Methodology

Sample preparation	For competition assays: RPE-1 TP53-/- dCas9-KRAB cells were infected at an MOI of ~0.2 and treated with 2µg/mL puromycin the next day and maintained in puromycin until the population was fully selected. This ensured that the transduced cell population all contained a puromycin resistance plasmid of NLS-mCherry LacZ-sgRNA or NLS-GFP GOI-sgRNA. Following selection, the mCherry- and GFP-expressing cells were mixed 1:1 (10,000 cells + 10,000 cells) and plated with or without drug in 12 well plate format. During the experiment, cells were subcultured and re-treated with or without drug every three days when they reached near-confluency. Samples were harvested by trypsinisation and resuspended in PBS for cytometric analysis.<br><br>For AHA incorporation assay: RPE-1 TP53-/- cells were seeded in a 6 well plate with a total volume of 2 mL medium per well. Wells were treated with or without drug the next day for 23 hours. Following this, the 6 wells were washed with PBS and treated for 1 hour in 2 mL of methionine free cell medium that contained the same drug concentrations as previous for each well. 50 µM L-Azidohomoalanine/AHA (Thermo, C10102) was added for 2 hours following a 1 hour methionine depletion. The 6 well plates were washed with PBS, typsinised and harvested. Following fixation, click reactions using Click-iT Cell Reaction Buffer (Thermo, C10269) and 2.5 µM Alexa Fluor 647 Alkyne, (Thermo, A10278) and subsequent washes were performed as per manufacturer instructions using the flow cytometry experimental protocol for adherent cells.
Instrument	BD Biosciences FACSymphony A5
Software	FlowJo v10.8.1
Cell population abundance	Cells were not sorted
Gating strategy	For competition assays, first, cells were gated based on FSC and SSC, and single cells were gated based on FSC-A and FSC-H. Gating for GFP and mCherry positive cells was determined using non-fluorescent and mono-fluorescent cells before analysing the mixed populations.<br><br>For AHA cells were also gated based on FSC and SSC, and single cells were gated based on FSC-A and FSC-H. Following this they were gated for the Alexa Fluor 647 intensity.

☒ Tick this box to confirm that a figure exemplifying the gating strategy is provided in the Supplementary Information.
